# A Decade-Long Delay in Presentation of Retained Foreign Bodies as Sequelae of Ballistic Injuries: A Report of Two Cases

**DOI:** 10.7759/cureus.77511

**Published:** 2025-01-15

**Authors:** Abira Chattopadhyay, Md Arif Hossain, Shrabasti Dey, Sweta Sikarwar, Inam Uddin

**Affiliations:** 1 Oral and Maxillofacial Surgery, Dr. R. Ahmed Dental College and Hospital, Kolkata, IND

**Keywords:** ballistic injury, case report, case report series, impacted bullet, retained foreign bodies, shrapnel, systemic toxicity

## Abstract

In many regions of the world, ballistic injuries are on the decline. With the reduced incidence of encountering these injuries, there is no general consensus on how to deal with ballistic fragments. Constant research in material science has led to the evolution of materials utilised in ballistics production. However, there has not been enough research into the effect of long-term exposure of the human body to these materials. Ballistic fragments, when asymptomatic and not in the vicinity of vital structures, are often thought to be innocuous in nature. Earlier concepts stated that these metallic fragments were encapsulated in the body by fibrosis and remained inert substances. However, the emerging evidence suggests that these retained ballistic fragments are more alike to ticking time bombs than the inert materials they were earlier thought to be. Side effects of long-term exposure to these materials can lead to not just systemic toxicity due to chronic metallic exposure but may even have carcinogenic effects.

This article reports two cases of retained ballistic fragments that presented in a delayed fashion due to their prior asymptomatic status coupled with inadequate investigation and intervention. It also discusses the issues associated with the retention of these fragments, especially in cases where adequate information is not available regarding the nature and composition of the retained materials or their interactions with the human body on long-term exposure.

## Introduction

Oral and maxillofacial surgery, as a speciality, owes its nascence to the massive carnage of the First and Second World Wars. Warring in the trenches led to a high volume of firearm and ballistic injuries to the head and neck region associated with complications such as infection, chronic pain, tissue damage, and even neurological impairment [1]. That, in turn, led to the necessity of the development of a speciality dedicated to the management of injuries in the highly specialised head and neck region with its myriad intricate structures integral to human existence and life experience. In today’s scenario, civilian oral and maxillofacial surgeons in various non-combat zones of the world are entirely unacquainted or rarely encounter ballistic injuries, except in theory. However, when encountered, the complex facial skeletal structure and the various vital structures lying in close proximity make management of these injuries a challenge, with both soft and hard tissues requiring appropriate management.

The categorisation of certain metals as human carcinogens in recent decades raises concerns about these fragments in addition to their physical presence as foreign entities and their propensity to produce systemic metal toxicity [2]. The physicochemical characteristics of metal-based nanoparticles, including their size, shape, chemical composition, and surface chemistry, determine their toxicity. In addition to the previously listed factors, nanoparticle toxicity is further influenced by solubility and agglomeration. However, metal-based nanoparticles' distinct physicochemical characteristics present unforeseen hazardous risks to the human body and encourage biological outcomes of metal toxicity [3].

Here we report two cases of delayed presentation of retained ballistic fragments managed at our institute where the patients presented with a complaint 9-13 years after the incidence of trauma.

## Case presentation

Case 1

A 31-year-old male patient reported to our department with a piece of metal protruding from the palate. The patient was one of the accused in an improvised explosive device (IED) attack nine years ago. He suffered multiple injuries which involved the presence of many embedded pieces of foreign material in various regions of the head and neck. Previously he had undergone two surgeries for retrieval of the foreign material. However, a comprehensive debridement had not been performed due to various constraints and bureaucratic red tape associated with the legal and judicial processes of healthcare for accused/convicted individuals leading to the persistent presence of metal debris.

According to the patient, he had noticed this foreign object only in the past few months. Initially, it seemed to be a small spicule. However, over time a large metallic object descended into his oral cavity and was impinging upon the gums and tongue due to which he wanted removal of the foreign body.

**Figure 1:**
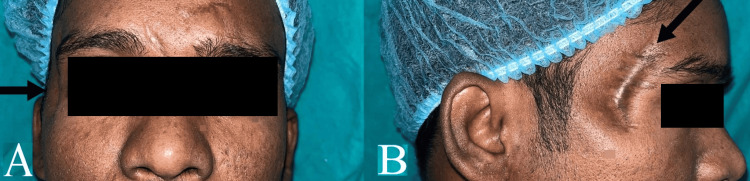
Case 1 - extraoral - (A) frontal view, (B) right lateral view - arrows indicate hollowing and scar adjacent to lateral rim of right orbit

A clinical examination of the patient revealed various scars on the forehead. There was a hollowing in the region adjacent to the lateral rim of the right orbit along with a surgical scar (Figure 1). The left eye displayed an antimongoloid slant associated with ptosis. On palpation, small nodular structures could be palpated in the right preauricular region suggestive of other embedded foreign bodies.

**Figure 2:**
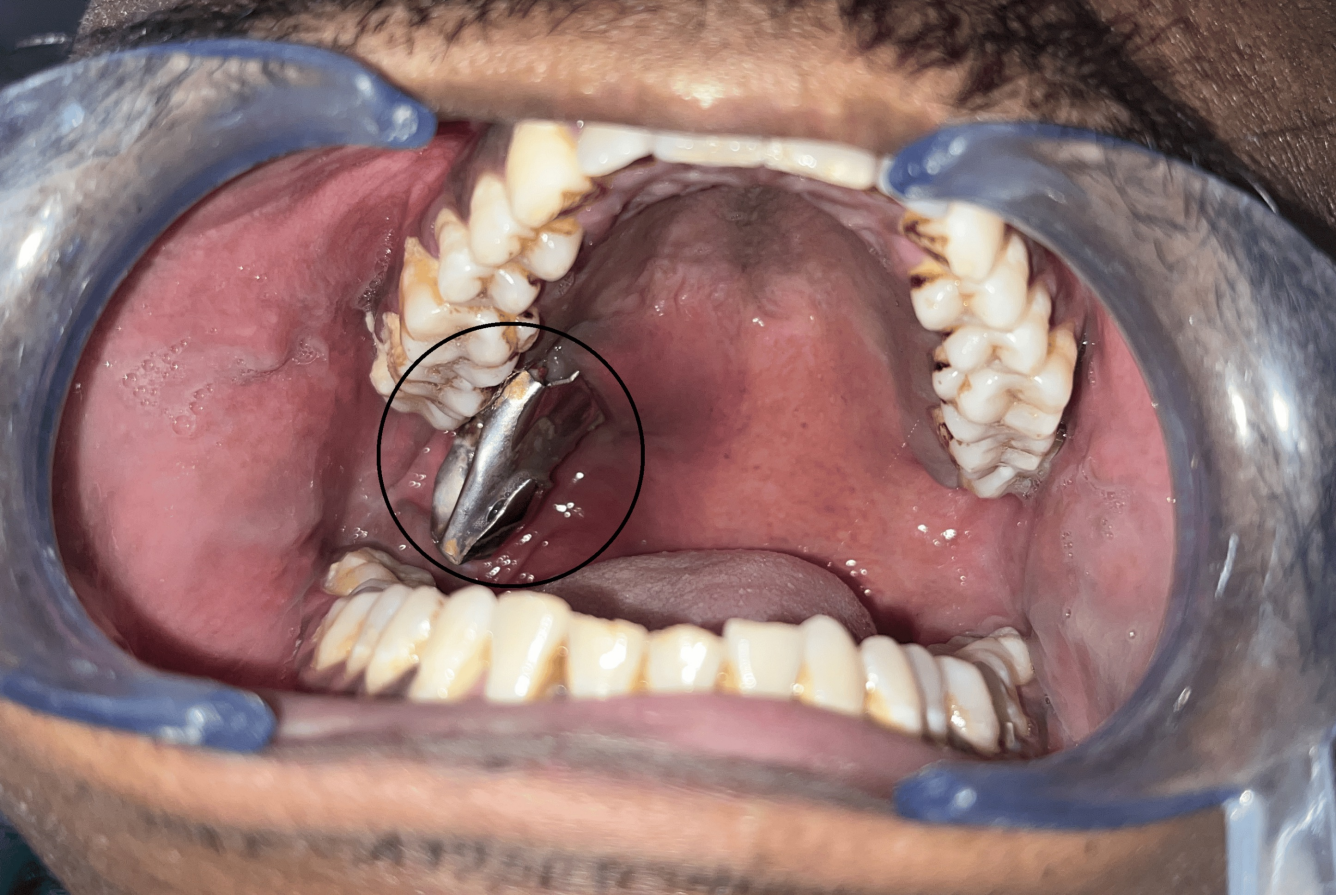
Case 1 - large metallic object protruding intraorally from palatal region adjacent to upper right third molar

Intraorally, a large metallic object was observed to protrude from the right maxillary tuberosity region at the junction of the hard and soft palate. Poor maintenance of oral hygiene was also observed (Figure 2). 

**Figure 3:**
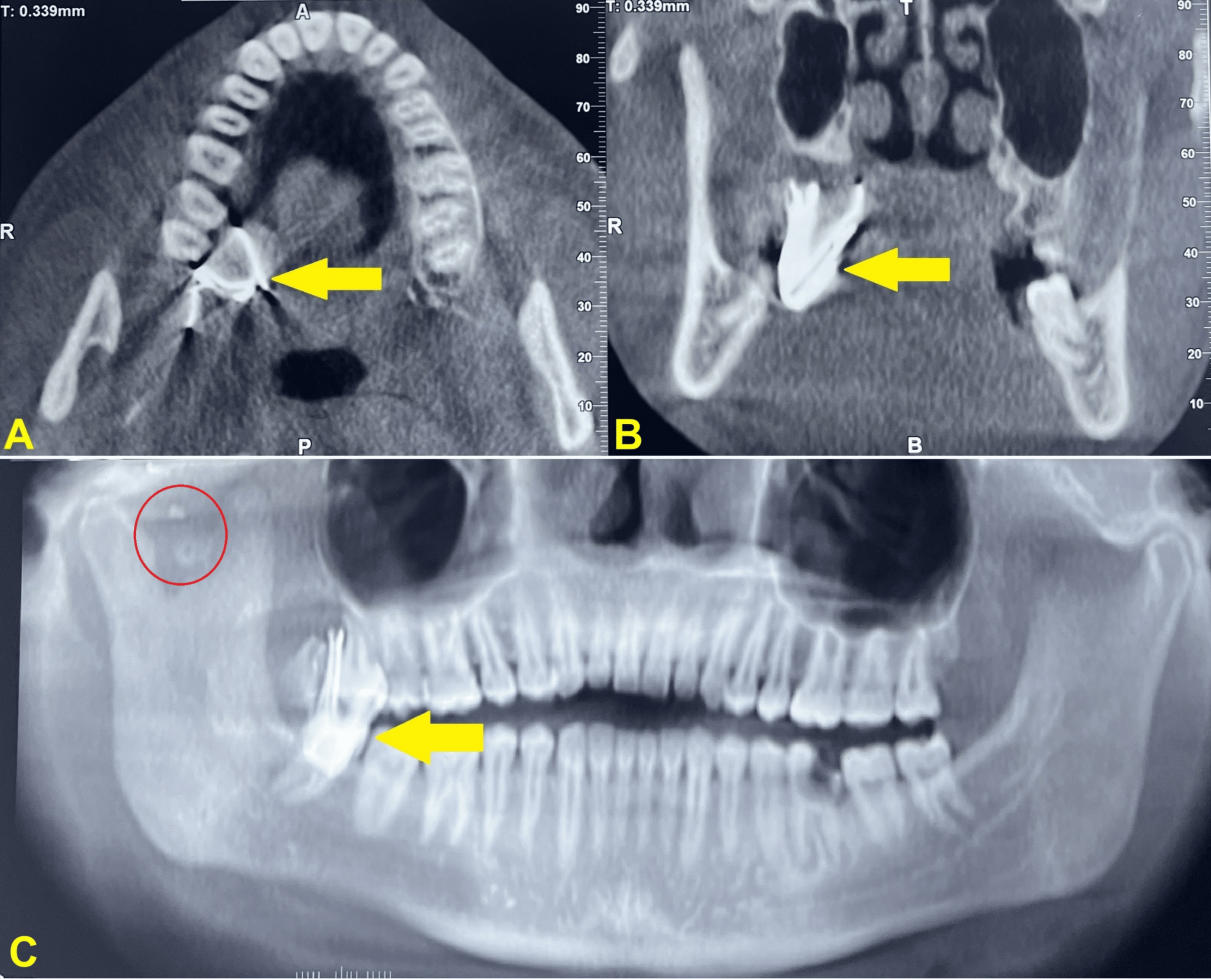
Case 1 - CBCT - (A) axial view, (B) coronal view, and (C) panoramic view show radiopacity in palatal region (yellow arrows). (C) Panoramic view also demonstrates small radiopaque particles in right preauricular region (red circle) suggestive of metallic foreign bodies CBCT - cone beam computed tomography

Cone beam computed tomography (CBCT) revealed a large well-defined radiopaque structure on the palatal aspect of the upper right second and third molar region which was embedded in the soft tissue of the soft palate along with smaller radiopaque structures in the right preauricular region between the condylar and coronoid processes. Scatter associated with the radiopaque structures suggested metallic foreign bodies (Figure 3).

**Figure 4:**
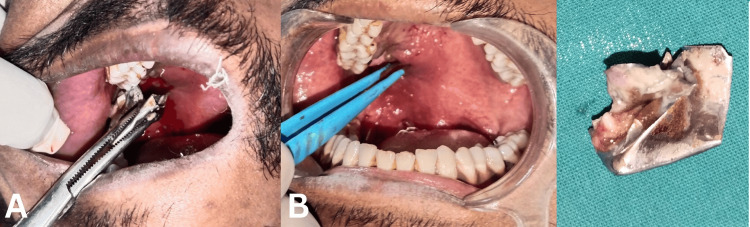
Case 1 - Ballistic fragment was removed from the palate after dissection around it (A) and hemostasis was achieved (B). The fragment was identified as scrap metal fragment (C)

Removal of the object was very straightforward and required very little soft tissue dissection around the ballistic fragment, after which it could be grabbed using artery forceps and removed from the soft tissue bed under local anaesthesia (2% lignocaine with 1:80,000 adrenaline, posterior superior alveolar nerve block along with local infiltration). Haemostasis of the residual defect was achieved using diathermy, sutures were not required. The defect was left to heal by secondary intention. On removal, the object was identified to be a scrap metal fragment (Figure 4).

**Figure 5:**
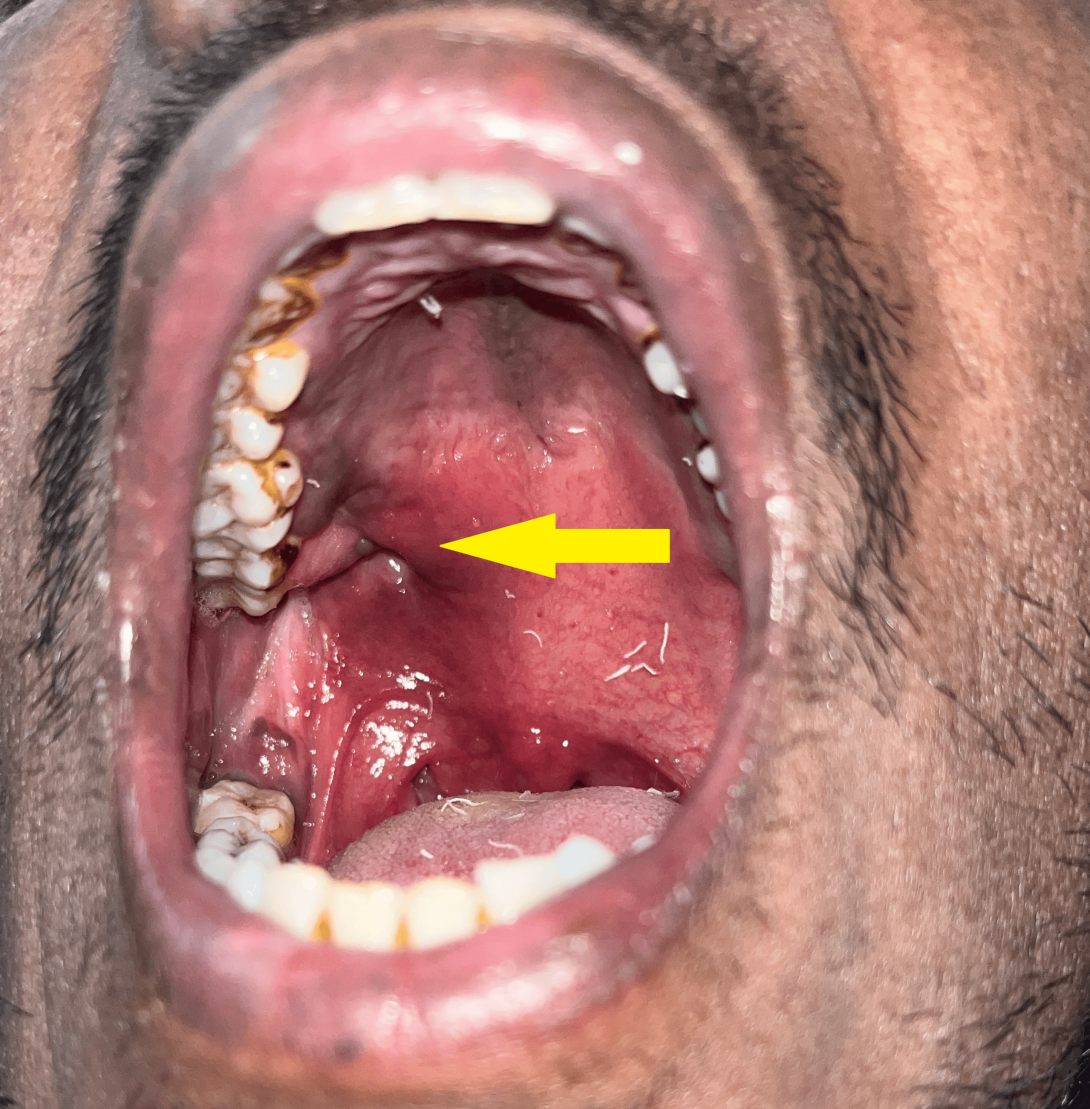
Case 1 - arrow indicates residual defect showing uneventful postoperative healing

Follow-up was done on the 7th and 14th postoperative days. Postoperative healing of the residual defect was uneventful and showed complete epithelialisation by the two-week mark (Figure 5).

Case 2

**Figure 6:**
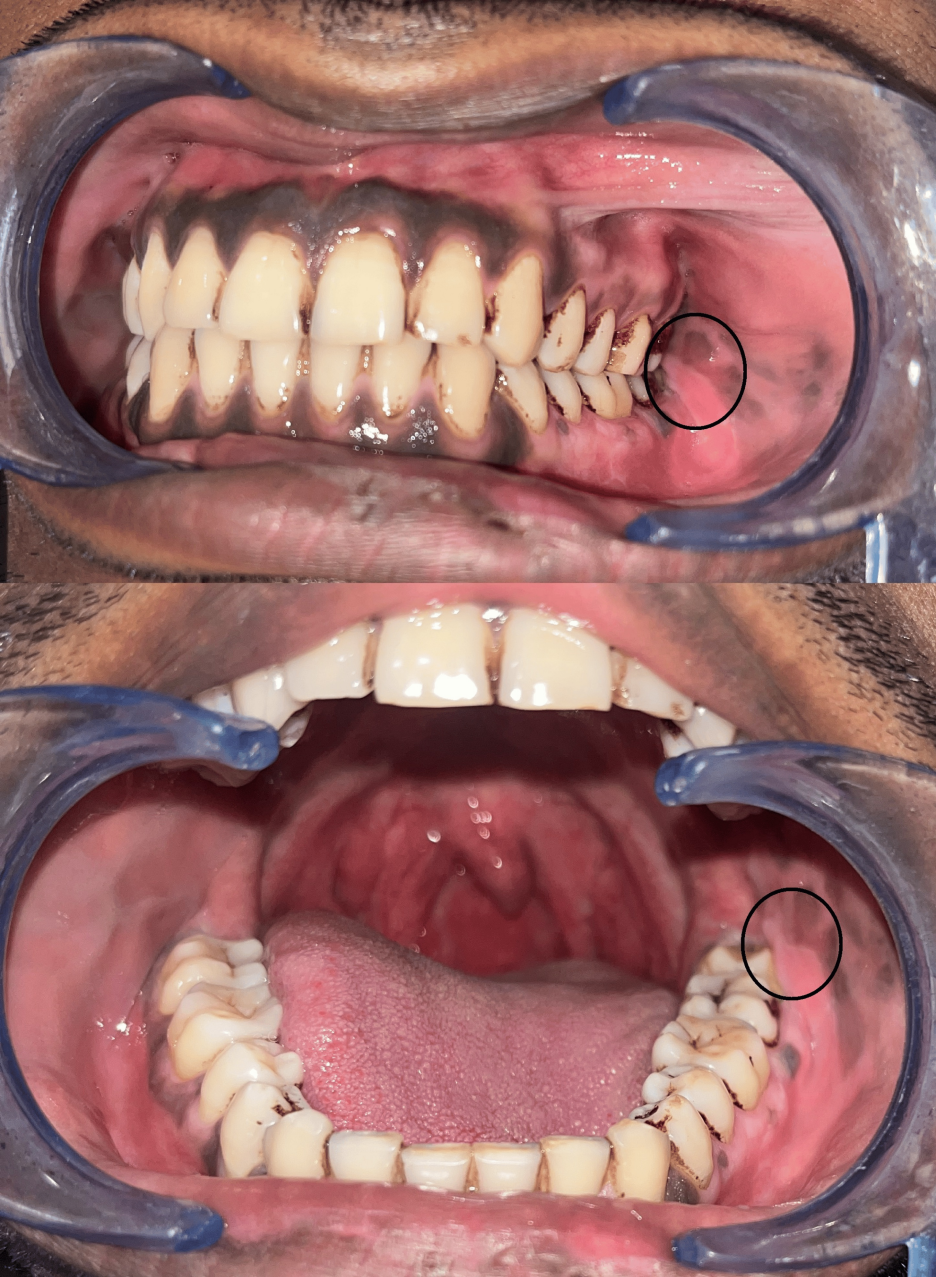
Case 2 - On palpation intraorally, irregular hard swelling was present in the circle-marked area

A 24-year-old male reported to our department with a complaint of dull pain in the posterior region of the lower jaw on the left side for the past month. The patient reported previous recurrent episodes of such pain in the same region over the last 2-3 years. However, he did not reveal any other significant history including pain or trauma to that region. On clinical examination, there were no significant extraoral findings. Intraoral examination revealed the presence of an impacted third molar with an overlying operculum in the lower left quadrant. There were no clinical signs of acute infection present intraorally. The gingiva displayed significant pigmentation barring a curious non-inclusion of the premolar-molar region of the lower left jaw. On palpation, an irregular hard swelling could be felt overlying the junction of the external oblique region and the body of the mandible which was tender on palpation (Figure 6). The patient was provisionally diagnosed with chronic pericoronitis and advised radiographic examination.

**Figure 7:**
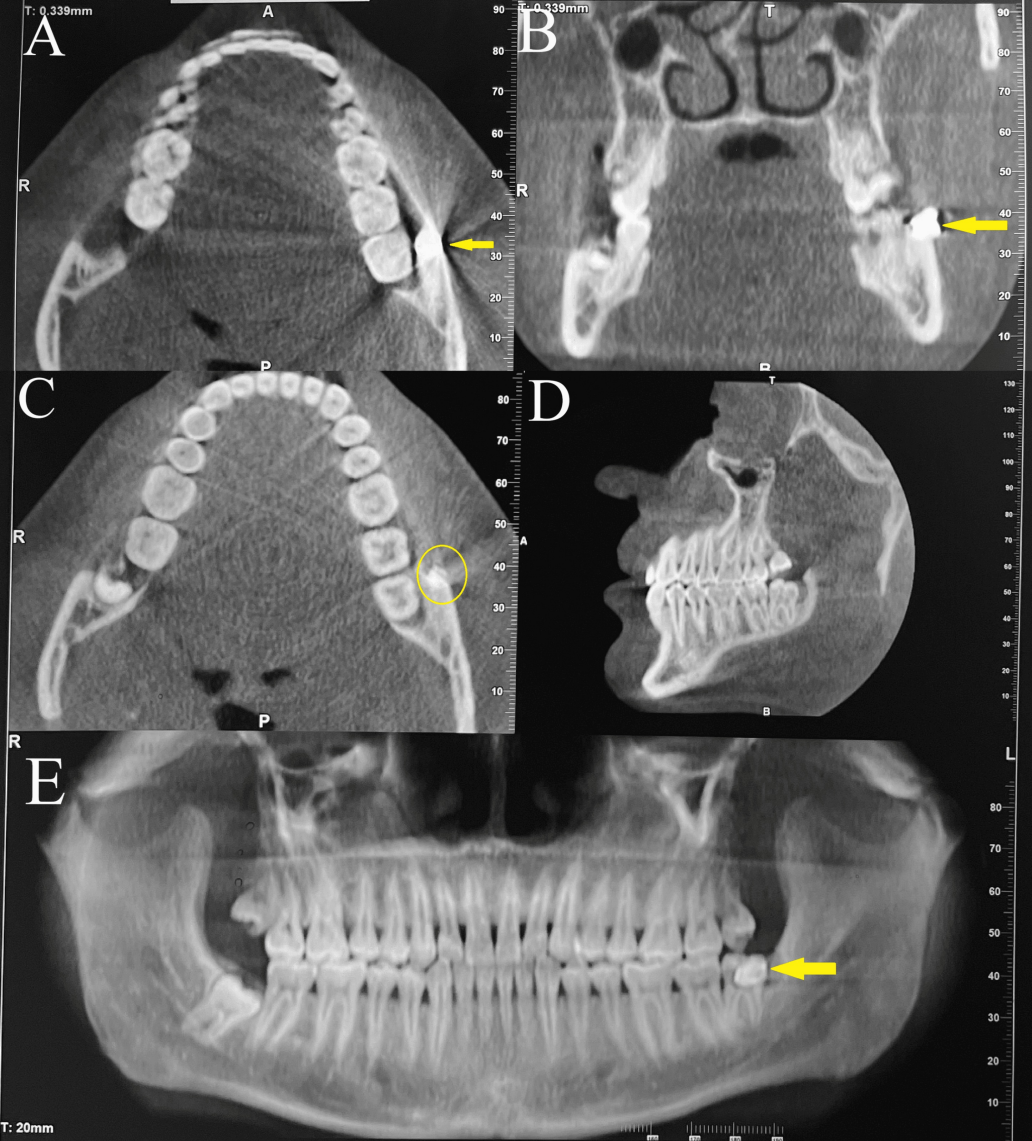
Case 2 - CBCT - (A,C) axial views, (B) coronal view, (D) sagittal view, and (E) panoramic view reveals well-defined radiopacity (indicated by yellow arrow/circle) exhibiting streak artifacts (A) on buccal aspect of lower left third molar region suggestive of metallic debris CBCT - cone beam computed tomography

On radiographic examination, CBCT revealed a radiopaque structure overlying the buccal cortical plate in the lower left third molar region. Streak artifacts were also noted arising from the same region leading to the suspicion of scatter from some metallic structure embedded in the bone of that region (Figure 7).

On further questioning to elicit relevant history in light of the radiographic findings, the patient revealed that around 13 years ago he had been accidentally shot by an air rifle while playing with friends in childhood. However, he had received only primary care. No surgical exploration or intervention was performed as there was uneventful healing. The patient was unaware of the presence of any remaining foreign body in that region.

**Figure 8:**
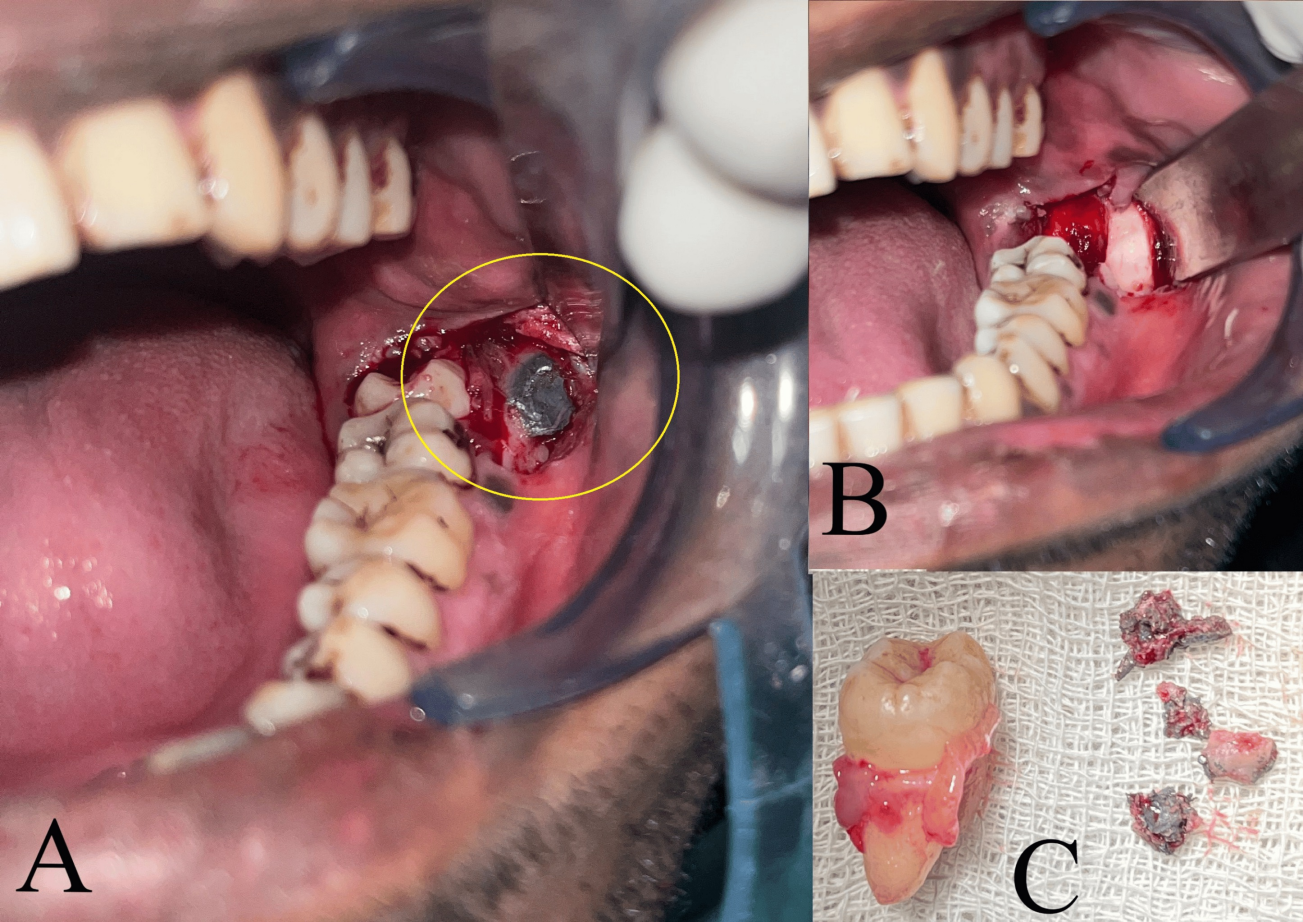
Case 2: (A) flap was raised to expose the foreign body; (B) foreign body and the third molar were removed; (C) extracted tooth and ballistic fragments

The patient underwent disimpaction of the impacted lower left third molar using Ward’s incision, followed by foreign body removal and debridement of the involved bone of the mandible via the same incision under local anaesthesia (2% lignocaine with 1:80,000 adrenaline, inferior alveolar nerve block). The metallic substance removed was partly flaky and partly clay-like in consistency (Figure 8). The incision was closed using 3-0 silk sutures that were removed after seven days. Adequate wound healing was noted within two weeks. Post-operative healing was uneventful and the patient reported resolution of pain after undergoing the surgery.

## Discussion

Ballistic wounds are loosely described as any injury attributable to firearms or explosives. Broadly they can be of two categories - those arising from bullets and those caused by fragmentation missiles and devices. The most common aetiologies for ballistic injuries are interpersonal violence and assault, as observed in the reported cases, along with accidents and suicide attempts, in that order [4]. Ballistic injuries can be: a) penetrating injuries caused by low-velocity projectiles that present with a bullet or fragment embedded in soft tissue associated with a small point of entry; b) perforating wounds caused by high-velocity projectiles that pass through the tissues with an entrance wound and a larger exit wound; in certain cases, the entrance wound is even noted to have healed before presentation for treatment; and c) avulsive wounds associated with loss of tissue [5].

These injuries may involve only soft tissue or both soft and hard tissues. Some may be associated with significant tissue loss, while some present without any loss of tissue. Depending on the location of injury and extent of penetration, they may also be potentially life-threatening or absolutely benign, thus resulting in a wide gamut of patterns of presentation. On initial presentation, the primary care consists of stabilisation of the patient depending upon the extent of injuries sustained. It is essential to ensure that any visible foreign body is left in situ as it is since they often act as a tamponade and hasty removal may initiate haemorrhage. Removal should be attempted only after adequate imaging with precise knowledge of the exact anatomic location and relation with surrounding structures [6].

As stated by Rowe and Williams, ballistic injuries to the head and neck differ from similar injuries to other parts of the body. The paucity of large muscle mass negates the possibility of damage from cavitation while the abundant blood supply enables healing even for dire injuries [5]. In a similar vein, in both the reported cases of penetrating injuries, the patients initially encountered rapid healing of the associated entry wound, which may be attributed to the extensive vascularity of the head and neck region promoting healing of local soft tissues. The prophylactic and empirical usage of antibiotics due to the high chance of bacterial contamination may also be attributed to this uneventful initial healing despite the retention of foreign debris in soft tissue. However, over the years, these patients have noticed extrusion of the foreign body (case 1) or pain associated with the retained debris (Case 2).

Foreign body that is retained in the body may often not be apparent on clinical examination or pose any discomfort to the patient leading to it being overlooked in the absence of detailed medical history. Thorough clinical and radiological examinations are also crucial for the detection of such cases. Diagnostic aids such as computed tomography (CT), magnetic resonance imaging (MRI), and ultrasonography, especially when the foreign body is lodged in soft tissue, are superior to routine radiographs for identifying and locating foreign bodies. For radiolucent objects, identification often hinges on the detection of indirect signs such as air bubbles, soft tissue oedema, or the formation of an abscess [6, 7].

The general consensus is that any foreign body should be removed to avoid infections, which may be either acute or chronic and associated with inflammation, discharge of pus, toxicity, impaired wound healing, or any immune response to the foreign body [6, 7]. Mechanical and functional impairment, neurological impairment, and compromised aesthetics are some other possible sequelae of the impacted foreign body which necessitate its removal. Removal of small foreign bodies may be performed under local anaesthesia, while larger objects or those that are more deeply embedded may require general anaesthesia. However, removal is not possible for all cases of the head and neck regions, where access is often compromised and the location is in close proximity to vital structures and where the risks of surgical exploration and retrieval of the foreign body outweigh the benefits. Often the retained foreign bodies exist uneventfully for years followed by delayed sequela many years later, as seen in both the reported cases [6]. According to Baum and associates, retained fragments are commonly those located in soft tissues which have limited accessibility, as they are swiftly enclosed by avascular scar tissue, reducing the risks of lead poisoning and infection in contrast to fragments retained in bone or intra-articular spaces [8].

In cases where retained shrapnel does not pose any significant discomfort to the patient and is clinically unapparent, it is imperative that the surgeon and patient are both aware of the presence of the retained foreign material and the potential risks associated with it. Signs and symptoms that seem unrelated at first glance can be properly evaluated for hazards such as metallic toxicity even in the absence of obvious signs, requiring surgical exploration and debridement only when the surgeon is aware of the presence of such substances. The previously established belief was that most embedded metallic shrapnel remained inert in the body, and surgical exploration for their removal carried a higher risk of damage to surrounding structures. However, as per Hoffman and associates, embedded metal fragments such as bullets and shrapnel from improvised explosive devices (IEDs) can lead to direct internalization of metals [9]. Contrary to earlier beliefs, current literature acknowledges the dearth of knowledge about the health effects of metal internalized as embedded shrapnel [10]. There are reports of cases where retained foreign bodies gave rise to various medical issues several years after the initial injury [9]. Animal studies performed by Kalinich and associates have reported significant carcinogenic effects of tungsten alloy-based munitions when implanted intramuscularly in rats [10]. Reports by Grasso and Short, as well as Constantini and associates, indicate systemic lead toxicity characterised by an elevated blood lead level resulting from retained shrapnel [11, 12]. The patients may be asymptomatic.

Symptoms, if present, are often non-specific, which makes it difficult to narrow down the cause to increased systemic levels of the offending metal without testing for its blood levels [12]. Although metallic toxicity due to chronic exposure from scattered metallic fragments is a well-established phenomenon, there is no consensus on the treatment of such cases [12].

Long-term exposure to metal fragments can result in metal ions being absorbed from tissue depots of metal pieces; systemic metal exposure has also been reported. Organ systems far from metal fragments embedded in soft tissue are also at risk of metal toxicity. Local tissues in proximity to fragments show signs of inflammatory processes and foreign‐body reactions, which can lead to carcinogenesis, which is, however, uncommon and dependent on the physical and elemental characteristics of the embedded material [2].

Skeletal muscles start showing changes from the initial six months. Histopathological analysis has revealed tumour development and inflammation that are characteristically distinct from areas that seem to be normal skeletal muscle tissue. Skeletal muscle also shows hypersensitivity reaction; high-persistent immune infiltration and inflammation are also seen in soft tissues [13]. Embedded fragments in hard tissue result in delayed healing and bone destruction.

Additionally, there are many challenges faced by healthcare providers during the treatment of these patients, including ethical and legal issues. Prior to treatment, proper consent in the patient’s own language in written and audio-visual recorded form is most important to avoid any medicolegal issues in the post-treatment period. Consent is the cornerstone of medical ethics and legal practice, emphasising a patient's right to autonomy and self-determination. Patients must be provided with sufficient and comprehensible information about the risks, benefits, and alternatives of proposed treatments. However, consent may not be required in life-threatening emergencies, as medical practitioners are bound by their duty to save lives [14]. Proper pre-operative diagnostic records like X-rays and ultrasounds should be maintained along with all findings upon examination for further reference. Confidentiality of the patient should also be maintained while taking pre-operative records. The patient should be informed about maintaining his/her pre-operative records for further reference if required.

## Conclusions

Ballistic injuries are highly individualised injuries that require individualised treatment plans taking into account various factors such as the type of munition involved, time of presentation, site of injury, as well as available resources, including the experience and expertise of the operating team. The lack of standard protocols for the management of embedded foreign bodies results in varying surgical outcomes dependent on the centre and surgeon involved. Age-old ideas of metallic fragments remaining inert and encapsulated upon fibrosis must be discarded. In light of the risks of heavy-metal toxicity from such retained metallic fragments, the aim should be the removal of all accessible fragments without jeopardizing the patient’s life as a consequence of the surgical intervention after a meticulous risk-benefit assessment.
